# Clinical significance of germline telomere length and associated genetic factors in patients with neuroblastoma

**DOI:** 10.1038/s41598-022-17246-4

**Published:** 2022-07-28

**Authors:** Joon Seol Bae, Ji Won Lee, Je-Gun Joung, Hee Won Cho, Hee Young Ju, Keon Hee Yoo, Hong Hoe Koo, Ki Woong Sung

**Affiliations:** 1 Research Institute for Future Medicine, Samsung Medical Center, Seoul, Republic of Korea; 2 Department of Pediatrics, Samsung Medical Center, Sungkyunkwan University School of Medicine, 81 Irwon-ro, Gangnam-gu, Seoul, 135-710 Republic of Korea; 3 Department of Biomedical Science, Cha Bundang Medical Center, Cha University, Seongnam, Republic of Korea

**Keywords:** Cancer, Genetics

## Abstract

Studies investigating the relationship between germline telomere length and the clinical characteristics of tumors are very limited. This study evaluated the relationship between germline telomere length and the clinical characteristics of neuroblastoma. In addition, a genome-wide association study (GWAS) was performed to investigate the genetic factors associated with germline telomere length. The germline telomere length of peripheral blood mononuclear cells from 186 patients with neuroblastoma was measured by quantitative polymerase chain reaction. The association between germline telomere length and clinical characteristics, including long-term survival, was investigated. For the GWAS, genotyping was performed with a high-density bead chip (Illumina, San Diego, CA, USA). After strict quality-control checks of the samples, an association analysis was conducted. The result showed that longer germline telomeres were significantly associated with longer event-free survival (*P* = 0.032). To identify significantly assocated genetic markers for germline telomere length, genome wide association analysis was performed. As a result, several single nucleotide polymorphisms located in *HIVEP3*, *LRRTM4*, *ADGRV1*, *RAB30*, and *CHRNA4* genes were discovered. During gene-based analysis (VEGAS2 tool), the *CNTN4* gene had the most significant association with germline telomere length (*P* = 1.0E−06). During gene ontology analysis, susceptible genes associated with germline telomere length were mainly distributed in neurite morphogenesis and neuron development. A longer germline telomere length is associated with favorable prognostic factors at diagnosis and eventually better event-free survival in patients with neuroblastoma. In addition, the GWAS demonstrated that genetic markers and genes related to germline telomere length are associated with neurite morphogenesis and neuron development. Further research with larger cohorts of patients and functional investigations are needed.

## Introduction

Telomeres are DNA–protein complexes at the ends of chromosomes that determine the lifespans of cells. As a repeat sequence (TTAGGC) present at the end of the chromosome, telomeres are known to play a role in preventing damage to the genome^1^. They protect chromosomes from end-to-end fusion^2^ and are involved in a variety of other functions, including cell death, cell senescence, abnormal cell proliferation, and separation during meiosis^3,4^. Telomere length has been reported to be inherited and has been found to vary between individuals of the same age^5^. Telomere length decreases when cells divide and, as telomere length decreases, cells age and die. Thus, telomere length has a profound relationship with age-related diseases and cancer^6–8^. Aging caused by telomere shortening differs from individual to individual and is known to be affected by environmental^9^ and genetic differences^10^. Recently, Degaldo et al. evaluated the familial inheritance of leukocyte telomere length by studying the association between identity-by-descent (IBD) shared at the end of chromosomes and the phenotypic similarity of leukocyte telomere length. They found that the leukocyte telomere length of parental germ cells affects the leukocyte telomere length of progeny cells and contributes to leukocyte telomere length heritability (*h*^2^) despite telomere "reprogramming" during embryonic development^11^. This implicates that leukocyte telomere length may be used as a genetic marker for disease susceptibility. In addition, Demanelis et al. found that long telomere lengths were present in all tissues in African ancestry. This finding supports the fact that telomere length is inherited through lineages. They also compared telomere lengths in various tissues using telomere length–related genomic variations and reported significant associations compared to whole-blood germline telomere length^12^.

Many genetic epidemiologic studies have reported results of the analysis of the association between germline telomere length and cancer risk^13–16^. A significant association of short germline telomere length with cancer risk in peripheral blood leucocytes and lung, bladder, head and neck, lung, renal cell, and breast cancer has been reported^13,14,17–19^. Functional studies using mouse models have shown that shorter germline telomere lengths are associated with an increased risk of cancers like epithelial and prostate cancer^14,20,21^. A shorter germline telomere length may promote cell aging and inhibit cancer progression. However, when the critical telomere length is reached, it causes genomic instability expansion and malignant transformation potential through the fusion bridge breakage cycle^22^. Short germline telomere lengths increase the risk of cancer for several reasons. Hongxia et al. performed a meta-analysis of 21 large-sample studies and reported that a short germline telomere length was associated with an increased cancer risk^17^. They suggested that the shortening of germline telomeres could reduce DNA repair capacity and cause complex cytogenetic abnormalities. In particular, the short germline telomere length in the specific chromosome arm could contribute to chromosome instability, which can lead to fatal aberrations, such as 1q, 8q, 17q, and 20q gains and 8p, 9p, 16q, and 17p losses. Conversely, conflicting results have also been offered, suggesting that longer germline telomere lengths increase the cancer risk^23–26^. A genetic study that measures the germline telomere length in various types of cancer for various populations and analyzes the association between germline telomere length and clinical characteristics of cancer patients is necessary to obtain more accurate conclusions. In particular, in the case of neuroblastoma, attempts to analyze the association between clinical features by measuring the telomere length of a cancer patient are very insufficient.

Telomere length, which is associated with chromosomal instability and thus the degree of cell protection from external stimuli, might also be associated with the clinical characteristics of tumors, including treatment outcomes. However, studies investigating the relationship between germline telomere length and the clinical characteristics of tumors are very limited in number. This study evaluated the relationship between germline telomere length and the clinical characteristics of neuroblastoma. In addition, a genome-wide association study (GWAS) was performed to investigate the genetic factors associated with telomere length.

## Methods

### Patients

A total of 186 patients diagnosed with neuroblastoma between May 2007 and July 2016 who had peripheral blood samples already cryopreserved at the Samsung Medical Center Biobank were enrolled in this study. This study was approved by the institutional review board (IRB) of Samsung Medical Center (IRB no. SMC 2015-11-053-035). Medical records were reviewed to obtain detailed clinical and biological data, such as the clinical presentation at diagnosis, tumor biology (including *MYCN* amplification status), tumor histology using the International Neuroblastoma Pathology Classification, and survival.

### Genome-wide genotyping

Genomic DNA was extracted from the collected blood using a Wizard Genomic DNA Purification Kit (Promega Corporation, Madison, WI, USA). DNA quantification was measured by fluorescence using Qubit equipment, and DNA integrity was checked using TapeStation equipment (Agilent Technologies, Santa Clara, USA). We used 200 ng of DNA that passed quality control for the Infinium assay (Illumina, San Diego, CA, USA). The chip used in this study was the Infinium Exome-24 BeadChip array (Illumina) containing 547,644 markers. The samples were processed according to the Infinium assay manual. Each sample was whole-genome–amplified, fragmented, precipitated, and resuspended in an appropriate hybridization buffer. The denatured samples were then hybridized on a prepared beadchip for a ≥ 16 h at 48 °C. Following hybridization, the bead chips were processed for the single-base extension reaction, staining, and imaging on an Illumina iScan system. The normalized bead-intensity data obtained for each sample were uploaded to the GenomeStudio software program (Illumina), which converted the fluorescent intensities into single-nucleotide polymorphism (SNP) genotypes. Sample quality was checked using a sample call rate of > 95%. In GenomeStudio, the cluster quality was measured using GenTrain scores, and then high-quality markers (> 0.7) were used.

### Germline telomere length measurement

Quantitative real-time polymerase chain reaction (qPCR) was used to measure germline telomere length^27^. The relative germline telomere lengths were measured as the ratio of the number of telomere (T) repeat copies to the number of single copy gene (S) copies (T:S ratio) in a given sample. Three replicates were performed on patient samples to calculate the average T:S ratio. The measurement of telomere length was performed by Mediage (Seongnam-si, Republic of Korea).

### Statistics

The clinical variables are summarized using mean ± standard deviation or median (range) values, as appropriate (Table 1). In the HelixTree software program (Golden Helix Inc., Bozeman, MT, USA), marker filtering was performed based on the following criteria: (1) call rate > 0.85, (2) minor allele frequency > 0.05, and Hardy–Weinberg equilibrium *P* value > 0.001. High-quality markers with values above those of the quality control criteria were used in the following analyses. For the GWAS, genotype distributions were compared using multivariable regression analyses in HelixTree. The figures of the Manhattan plot and regional association plots were drawn by R (R Foundation for Statistical Computing, Vienna, Austria) and the LocusZoom tool (Center for Statistical Genetics, Department of Biostatistics, University of Michigan, Ann Arbor, MI, USA), respectively. The gene-based analysis was performed using the VEGAS2 tool (QIMR Berghofer Medical Research Institute, Herston, Australia). Among the VEGAS2 options, we choose 1000 G Asians for SNPs and all Asians for sub-population. The analysis was carried out for 3 groups: all and 10% and 20% extreme groups. A gene-pathway analysis was done using VEGAS2. The event-free survival (EFS) and overall survival (OS) rates were estimated using the Kaplan–Meier method, and differences in survival curves were compared with the log-rank test. A multivariate analysis for EFS was performed by Cox regression analysis. Clinical characteristics were compared between 2 groups using the Pearson chi-squared test or Fisher’s exact test for categorical variables and the *t* test or Kruskal–Wallis rank-sum test for continuous variables. *P* < 0.05 was considered to be statistically significant.

**Table 1:** Germline telomere length according to clinical characteristics.

Clinical Characteristics	No. (%)	Median (range)	*P*-value
**Sex**			0.820
Male	99 (53.2)	17.51 (8.28–29.36)	
Female	87 (46.8)	17.18 (8.41–26.73)	
**Age at diagnosis**			0.113
< 1.5 years	73 (39.2)	18.05 (8.41–29.36)	
> 1.5 years	113 (60.8)	17.22 (8.28–25.17)	
**Primary site**			**0.015**
Abdomen	141 (75.8)	17.18 (8.41–27.46)	
Others	45 (24.2)	18.78 (8.28–29.36)	
**Stage**			**0.007**
1, 2	50 (26.9)	18.44 (8.28–29.36)	
3, 4, 4S	136 (73.1)	17.11 (8.41–25.70)	
**Differentiation (N = 166)**			0.891
GNB	39 (23.5)	18.00 (8.28–25.17)	
Differentiating	37 (22.3)	17.85 (9.93–25.10)	
PD/UD	90 (54.2)	17.65 (8.48–29.36)	
**MYCN amplification (N = 183)**			**0.046**
Absent	155 (84.7)	17.85 (8.28–29.36)	
Present	28 (15.3)	16.20 (8.48–24.73)	
**1p deletion (N = 99)**			0.856
Absent	86 (86.9)	18.41 (11.07–27.46)	
Present	13 (13.1)	17.80 (15.20–25.17)	
**11q deletion (N = 98)**			0.893
Absent	73 (74.5)	18.57 (11.07–27.46)	
Present	25 (25.5)	18.10 (12.72–25.70)	
**17q gain (N = 97)**			0.300
Absent	66 (68.0)	18.41 (11.07–27.46)	
Present	31 (32.0)	18.46 (15.41–25.70)	
**Risk group**			**0.042**
Low	50 (26.9)	18.36 (8.28–29.36)	
Intermediate	49 (26.3)	17.18 (8.41–25.70)	
High	87 (46.8)	17.44 (8.28–29.36)	

Abbreviations: Ganglioneuroblastoma (GNB), poorly differentiated (PD), undifferentiated (UD).

Stage 1, 2, and 4S tumors were stratified into the low-risk group if MYCN was not amplified, whereas stage 4 tumors in patients older than 12 months (until 2008) or 18 months (since 2009) or any tumors with amplified MYCN were classified as the high-risk group. The intermediate-risk group includes all other tumors not mentioned above.

## Results

### Clinical characteristics and their association with germline telomere length

**Figure 1:**
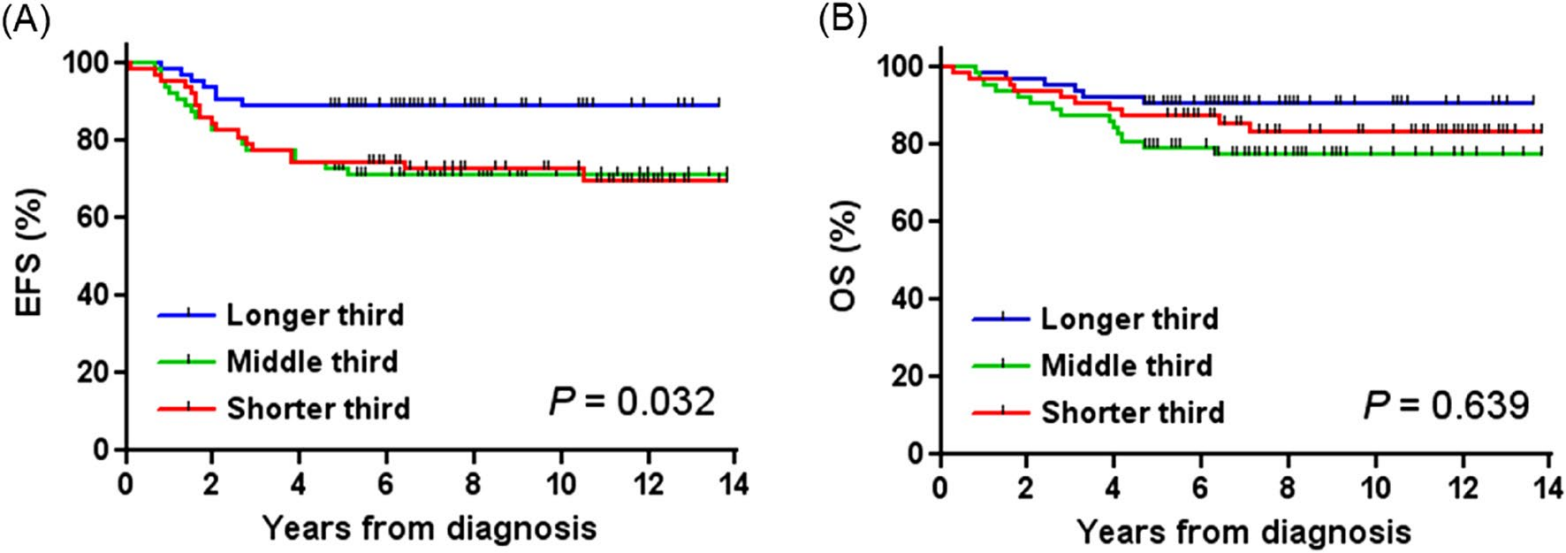
Survival data according to germline telomere length. (**A**) Patients with longer third telomeres showed longer event-free survival than other patients, (**B**) but there was no statistical difference in overall survival.

The clinical characteristics of the 186 study participants are given in Table 1. The median age at diagnosis was 2.1 years (range, 0.0–19.3 years), and 87 (46.8%) patients were categorized into the high-risk group. We tested the association between germline telomere length and the following 10 important clinical characteristics in neuroblastoma: sex, age at diagnosis, primary site, tumor stage, tumor differentiation, *MYCN* amplification, 1p deletion, 11q deletion, 17q gain, and risk group. We found that an extra-abdominal primary site, lower stage (stage 1/2), *MYCN* non-amplified tumor, and low-risk group categorization were associated with a longer germline telomere length (*P* = 0.015, 0.007, 0.046, and 0.042, respectively). Patients with longer third telomeres had longer EFS than other patients (*P* = 0.032), but there was no such difference in OS (Fig. 1). Table 2 lists the results of the multivariate analysis for EFS. An age at diagnosis of > 1.5 years (hazard ratio, 3.08; *P* = 0.017), stage 4 (hazard ratio, 4.75; *P* = 0.003), and longer third telomere length (hazard ratio, 0.037; *P* = 0.046) were independent prognostic factors for EFS.

**Table 2:** Multivariate analysis for event-free survival (EFS).

Risk factors	Hazard ratio (range)	*P*-value
Age at diagnosis > 1.5 years	3.08 (1.22–7.78)	**0.017**
Stage 4	4.75 (1.70–13.26)	**0.003**
MYCN amplification	1.40 (0.65–3.03)	0.392
**Differentiation**		0.456
GND	1.00	
Differentiating	0.88 (0.28–2.85)	0.840
PD/UD	1.46 (0.53–4.00)	0.466
**Germline telomere length**		0.131
Shorter third	1.00	
Middle third	0.71 (0.36–1.42)	0.338
Longer third	0.37 (0.14–0.98)	**0.046**

### Genetic association analysis of germline telomere length

**Figure 2:**
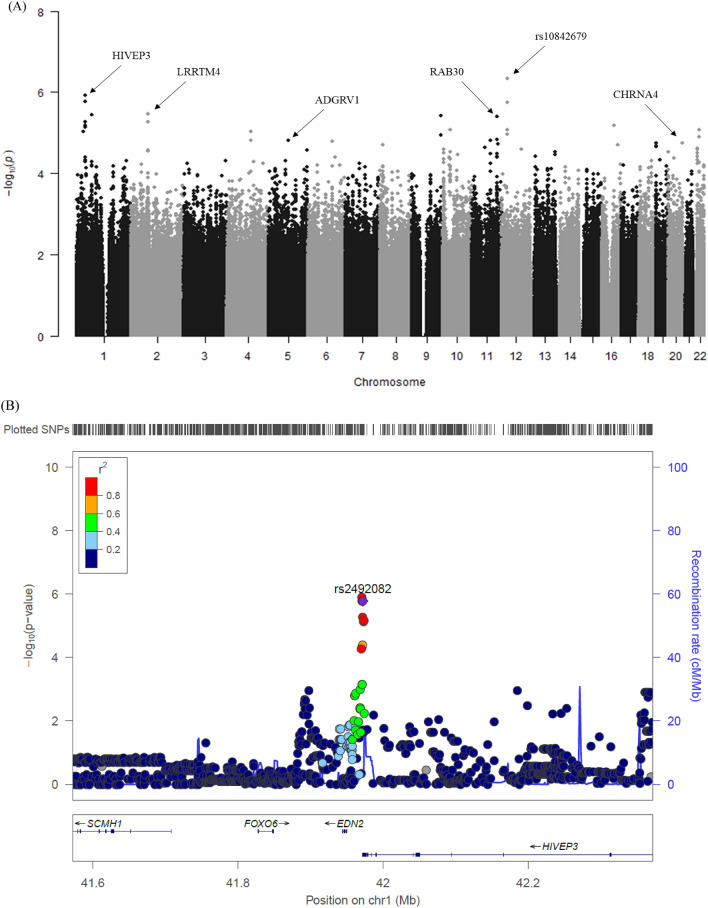
Manhattan plot and regional association plot. (**A**) *P* values from the genome-wide association study. The Manhattan plot shows the *P* values for the risk of neuroblastoma calculated using a logistic regression analysis. The X-axis represents the single-nucleotide polymorphism (SNP) markers on each chromosome. The highest *P* value (*P* = 4.7E-07) was observed for rs10842679 on 12p.12.1. (**B**) Regional association plots at *CNTN4*. Regional association plots containing both genotypes and SNPs within 400 kb of *CNTN4* were generated by LocusZoom. The significance of the association (− log10-transformed *P* values) and recombination rate is plotted. SNPs are colored to reflect pairwise linkage disequilibrium (*r*^2^) with the most significantly associated genotyped SNPs in the 1000 Genomes Project Phase 1 interim release Asian (ASN) population genotypes. The most significant genotyped SNPs are labeled and shown in purple.

We tested associations with germline telomere length in neuroblastoma patients in a multivariable regression analysis using age and sex as covariates. After marker filtering, 248,399 markers were used in the association analysis. We found several significant markers associated with germline telomere length within the *HIVEP3*, *LRRTM4*, *ADGRV1*, *RAB30*, and *CHRNA4* genes (Fig. 2A). The significant markers for germline telomere length are summarized in Table 3. In the GWAS, the most highly associated marker was rs10842679 (*P* = 4.7E-07). The gene nearest rs10842679 was *BHLHE41*, which is known as a putative regulator of neuronal differentiation^28^. Interestingly, markers located in the 3’UTR of the *HIVEP3* gene showed a strong association with germline telomere length (Table 3). The germline telomere length in the markers tended to increase from the major allele to the minor allele. We analyzed the regional associations of 400 kb around *HIVEP3* on chromosome 1p34.2 (Fig. 2B) and found that the rs2492082 marker had a relatively robust association signal (*P* = 1.7.E-06) (Table 3, Fig. 2B). To investigate the effects of multiple SNPs on germline telomere length in our neuroblastoma patients, we performed a gene-based assay using the VEGAS2 algorithm^29^. We found that the *CNTN4* gene had the most significant association (*P* = 1.0E-06). The results of the gene-based analysis are summarized in Supplementary Table 1.

**Table 3:** Single nucleotide polymorphisms associated with germline telomere length in neuroblastoma patients.

SNP	CHR	BP	Alleles	Gene	Region	MAF	DD* (TL)	Dd* (TL)	dd* (TL)	*P*-value
rs10842679	12	26281858	G > C			0.084	155 (16.6)	29 (20.1)	1 (25.2)	4.7E−07
rs10890075	1	41970768	T > C			0.395	67 (15.6)	90 (17.7)	28 (19.5)	1.2E−06
rs11210339	1	41971212	A > G			0.392	68 (15.6)	89 (17.7)	28 (19.5)	1.7E−06
rs2492082	1	41972198	T > A	HIVEP3	UTR3	0.392	68 (15.6)	89 (17.7)	28 (19.5)	1.7E−06
rs1383116	12	26278079	A > C			0.081	156 (16.7)	28 (20.0)	1 (25.2)	1.8E−06
rs71420970	2	77012296	C > G	LRRTM4	intron	0.070	159 (16.7)	26 (20.5)		3.5E−06
rs6679278	1	70851205	G > A			0.254	106 (16.1)	64 (18.5)	15 (19.6)	3.6E−06
rs11243436	9	134449467	C > T			0.100	152 (16.6)	29 (19.6)	4 (22.3)	3.8E−06
rs11243437	9	134450073	C > G			0.100	152 (16.6)	29 (19.6)	4 (22.3)	3.8E−06
rs12806316	11	113495279	C > A			0.414	63 (15.4)	91 (17.9)	31 (18.9)	3.9E−06
rs2492080	1	41971867	A > C			0.395	67 (15.7)	90 (17.7)	28 (19.3)	5.3E−06
rs35359723	2	77015092	G > A	LRRTM4	intron	0.065	161 (16.7)	24 (20.6)		5.5E−06
rs2731765	16	58276591	C > A			0.300	87 (18.2)	85 (16.8)	13 (12.9)	6.5E−06
rs2257931	16	58279244	A > G			0.300	87 (18.2)	85 (16.8)	13 (12.9)	6.5E−06
rs1969749	1	41973908	T > C	HIVEP3	UTR3	0.405	63 (15.7)	94 (17.5)	28 (19.6)	6.6E−06
rs2810587	1	41973095	G > A	HIVEP3	UTR3	0.400	64 (15.7)	94 (17.6)	27 (19.4)	7.3E−06
rs1383112	12	26287230	T > C			0.092	152 (16.7)	32 (19.6)	1 (25.2)	8.4E−06
rs1383113	12	26287240	A > G			0.092	152 (16.7)	32 (19.6)	1 (25.2)	8.4E−06
rs2729628	12	26290639	T > C			0.092	152 (16.7)	32 (19.6)	1 (25.2)	8.4E−06
rs1780432	10	34184022	C > T			0.208	112 (18.2)	69 (15.9)	4 (13.3)	8.5E−06
rs1740718	10	34184861	C > A			0.208	112 (18.2)	69 (15.9)	4 (13.3)	8.5E−06
rs956613	10	34185869	G > C			0.208	112 (18.2)	69 (15.9)	4 (13.3)	8.5E−06
rs74386538	22	27516762	A > G			0.257	101 (18.2)	73 (16.4)	11 (13.7)	8.6E−06
rs417693	4	111211272	G > A			0.124	140 (16.5)	44 (19.2)	1 (24.4)	9.2E−06
rs10915048	1	30682811	A > G			0.254	99 (16.1)	78 (18.4)	8 (20.0)	9.2E−06
rs3809140	12	26278444	G > A			0.089	153 (16.7)	31 (19.6)	1 (25.2)	1.1E−05
rs11243434	9	134448487	G > A			0.097	153 (16.7)	28 (19.5)	4 (22.3)	1.1E−05
rs80083893	22	27516984	G > A			0.265	99 (18.2)	74 (16.4)	12 (13.9)	1.3E−05
rs12788951	11	113471957	T > C			0.416	63 (15.4)	90 (18.0)	32 (18.7)	1.5E−05
rs7931613	11	82694032	T > C	RAB30	intron	0.403	63 (16.0)	95 (17.2)	27 (20.0)	1.5E−05

Abbreviation: CHR (chromosome), BP (base pair), TL (telomere length), D (dominant allele), d (recessive allele),

*DD, Dd, dd means GG, GC, CC genotypes in rs10842679 (Alleles: G > C).

## Discussion

In this study, we investigated the association between germline telomere length in peripheral blood mononuclear cells, not tumor cells, and the clinical characteristics of tumors at diagnosis. Patients with an extra-abdominal primary tumor, lower-stage tumor, *MYCN* non-amplified tumor, or low-risk tumor had longer germline telomere lengths than other patients. These clinical features are usually associated with a better prognosis, and thus, it was unsurprising that a longer germline telomere length was associated with better EFS and was an independent prognostic factor for EFS in our multivariate analysis. This is the first study to elucidate the clinical significance of germline telomere length in patients with neuroblastoma. We cannot explain the reason for the association between germline telomere length and the clinical characteristics of tumors. However, our results suggest that germline genomic characteristics, including germline telomere length, might affect the clinical characteristics of tumors at diagnosis and the treatment response.

For this reason, we performed genome-wide genotyping with a high-density bead chip to identify the genetic factors related to germline telomere length. In the GWAS analysis, novel risk SNPs, including *HIVEP3* and *LRRTM4*, were significantly associated with germline telomere length, although the results did not reach significance with Bonferroni correction (Table 3). That most significant marker rs10842679 was adjacent to the *BHLHE41* (basic helix-loop-helix family member e41) gene. *BHLHE41* is known to be a transcription factor implicated in cellular functions such as proliferation, differentiation, and tumorigenesis^28^. It is possible that this marker is linked to genetic factors that directly or indirectly affect the expression of the *BHLHE41* gene. Follow-up functional studies would be required to clarify the exact mechanism. Qin et al. reported that the marker showed higher levels of *HIVEP3* and *SOX9* messenger RNA expression than non-carcinoma cells^30^. In particular, patients with *HIVEP3* and *SOX9* overexpression showed a lower survival rate. Follow-up studies comparing therapeutic effects and survival rates according to the expression of the *HIVEP3* gene and germline telomere length changes are needed to clarify how germline telomere length is related to the function of the *HIVEP3* gene.

In a gene-based analysis using the VEGAS2 tool, the *CNTN4* gene showed the most significant association. The *CNTN4* gene encodes contactin 4, a member of the immunoglobulin superfamily^31^. The functions of proteins in this family are suggested to involve synaptic plasticity. In addition, they are known to be involved in axon growth, guidance, and fasciculation^32^. Although the details remain unclear, the effect of *CNTN4* on the development of nerve endings and the development of neuroblastoma should be examined in future functional studies. In a Gene Ontology analysis, the statistically significant categories (empirical *P* < 0.00005) were cadherin binding, cell-to-cell pathway, cell-leading edge, neurite morphogenesis, cell-part morphogenesis, and neuron development.

Shortened germline telomere lengths were associated with the risk of cancer. Hongxia et al. reported that a short germline telomere length was associated with an increased cancer risk after investigating many related publications^17^. In addition, Walsh et al. also reported common genetic polymorphisms associated with longer telomere length in the risk of childhood cancers, including neuroblastoma^33^. They suggested that many genetic loci with a weak effect may contribute to impact telomere biology and neuroblastoma risk. This suggestion explains the reason why more genetic variants should be investigated in the relationship with germline telomere length in various populations, including Asians. This is the first GWAS study to investigate an association with germline telomere length in neuroblastoma patients, and we found that the *CNTN4* gene is associated with changes in germline telomere length in Korean neuroblastoma patients. Our number of samples was insufficient due to the rarity of neuroblastoma. However, the new genes and markers discovered through this study will contribute to other GWAS studies that can be conducted in various ethnicities in the future.

In conclusion, we found that a longer germline telomere length is associated with favorable prognostic factors at diagnosis and eventually a better EFS among patients with neuroblastoma. In addition, the GWAS demonstrated that genetic markers and genes related to germline telomere length were associated with neurite morphogenesis and neuron development in neuroblastoma. Further studies with larger cohorts of patients and functional investigations are needed.

## Supplementary Information

Supplementary Information.

## Data Availability

The datasets generated or analyzed during the current study are not publicly available due to a prohibition of external release of patient-derived data but may be available from the corresponding author upon reasonable request through strict deliberation by the Data Deliberation Committee.
