# Supplementary material for: Clinical significance of germline telomere length and associated genetic factors in patients with neuroblastoma
Source: Sci Rep. 2022 Jul 28;12:12954. doi: 10.1038/s41598-022-17246-4 (PMC9334347; doi:10.1038/s41598-022-17246-4)
Supplement: Supplementary file 1 — Supplementary Information. [file 41598_2022_17246_MOESM1_ESM.docx]

Supplementary Table 1. Result of gene-based analysis with telomere length using VEGAS2

| Chromosome | Gene | Number of SNPs | Start(bp) | Stop(bp) | *P*-value | Top SNP |
| --- | --- | --- | --- | --- | --- | --- |
| 3 | CNTN4 | 56 | 2140549 | 3099645 | 1.0E-06 | rs71311728 |
| 2 | CTNNA2 | 44 | 79740059 | 80875988 | 1.0E-06 | rs2974167 |
| 11 | NELL1 | 45 | 20691096 | 21597229 | 1.0E-06 | rs75333101 |
| 18 | KCTD1 | 3 | 24034873 | 24237365 | 1.0E-06 | rs3898814 |
| 18 | L3MBTL4 | 23 | 5954704 | 6414910 | 1.0E-06 | rs1784821 |
| 16 | RBFOX1 | 9 | 6069131 | 7763340 | 1.0E-06 | rs6500837 |
| 7 | CNTNAP2 | 30 | 145813452 | 148118088 | 1.0E-06 | rs10264551 |
| 11 | NAV2 | 39 | 19372270 | 20143147 | 2.0E-06 | rs7110043 |
| 11 | NTM | 25 | 131240370 | 132206716 | 3.0E-06 | rs11222598 |
| 21 | MIR5009 | 10 | 28659821 | 29283529 | 3.0E-06 | rs76860190 |
| 2 | EML6 | 49 | 54952148 | 55199156 | 6.0E-06 | rs2631839 |
| 2 | LRRTM4 | 118 | 76974849 | 77749502 | 7.0E-06 | rs71420970 |
| 10 | CTNNA3 | 112 | 67672275 | 69455949 | 8.0E-06 | rs12571490 |
| 7 | ELMO1 | 38 | 36892510 | 37488929 | 8.0E-06 | rs11770274 |
| 8 | CSMD1 | 116 | 2792874 | 4852328 | 9.0E-06 | rs10106035 |

*bp: base pair

Supplementary Table 2. Result of gene ontology analysis for telomere length

| GO ID | Pathway *P* | Empirical *P* | Gene list |
| --- | --- | --- | --- |
| GO:0045296_cadherin_binding | 2.15E-16 | 0.00005 | CTNNA2_CTNNA3_CDH13_PTPRM_PTPRT |
| BIOCARTA_CELL2CELL_PATHWAY | 2.98E-10 | 0.00007 | CTNNA2_CTNNA3 |
| GO:0031252_cell_leading_edge | 2.07E-31 | 0.0002 | SCYL3_CDC42BPA_ANTXR1_CTNNA2_FGD5_PDLIM7_CNTNAP2_MTSS1_CTNNA3_CDC42BPB_IQGAP1_GAS7_PTPRM_NF2 |
| GO:0048812_neurite_morphogenesis | 3.77E-67 | 0.0005 | EPHB2_NTNG1_LMX1A_NFASC_NRXN1_CTNNA2_NRP2_KLF7_CNTN4_TOP2B_EPHB1_SLIT2_UNC5C_SEMA6A_SLIT3_EGFR_SEMA3A_NRCAM_PARD3_DSCAML1_PVRL1_FEZ1_MYCBP2_NRXN3_BCL11B_IGF1R_RTN4RL1_GAS7_PRKCA_PTPRM_AFG3L2_DCC_CACNA1A_SNAP25_CDH4_APP_DSCAM |
| GO:0032990_cell_part_morphogenesis | 1.57E-68 | 0.0006 | EPHB2_NTNG1_LMX1A_NFASC_NRXN1_ALMS1_CTNNA2_NRP2_KLF7_CNTN4_TOP2B_EPHB1_SLIT2_UNC5C_SEMA6A_SLIT3_EGFR_SEMA3A_NRCAM_PARD3_DSCAML1_PVRL1_FEZ1_MYCBP2_NRXN3_BCL11B_IGF1R_RTN4RL1_GAS7_PRKCA_PTPRM_AFG3L2_DCC_CACNA1A_SNAP25_CDH4_APP_DSCAM |
| GO:0048666_neuron_development | 4.38E-79 | 0.0007 | TP73_EPHB2_OLFM3_NTNG1_LMX1A_NFASC_NRXN1_ALMS1_CTNNA2_NRP2_KLF7_CNTN4_TOP2B_EPHB1_SLIT2_UNC5C_GPR98_SEMA6A_SLIT3_TULP1_CLIC5_SEMA3A_NRCAM_CNTNAP2_PARD3_PRKG1_MYO7A_DSCAML1_PVRL1_FEZ1_OPCML_MYCBP2_NRXN3_BCL11B_IGF1R_RTN4RL1_PRKCA_PTPRM_AFG3L2_DCC_CACNA1A_SNAP25_CDH4_APP_DSCAM |
| GO:0031225_anchored_to_membrane | 1.11E-33 | 0.0008 | NEGR1_NTNG1_GPC1_CNTN4_CNTN3_GPIHBP1_ART1_DLG2_CNTN5_NCAM1_OPCML_GPC6_DNAJA4_CDH13_RTN4RL1_SNAP25 |
| PANTHER_MOLECULAR_FUNCTION_Transcription_factor | 9.43E-29 | 0.0008 | NFIA_ESRRG_RARB_MAML3_NFIB_MLLT3_MAML2_ESR2_RORA_GAS7_L3MBTL4_LZTR1_TCF20 |
| PANTHER_BIOLOGICAL_PROCESS_Cell_adhesion | 5.29E-123 | 0.0008 | COL16A1_PTPRF_NEGR1_IGFN1_NFASC_LAMB3_NRXN1_PKP4_PARD3B_FN1_TNS1_AGAP1_GPC1_CNTN4_CNTN3_CLSTN2_NLGN1_PCDH7_TSPAN5_FAT4_CTNND2_BAI3_COL12A1_CD164_LAMA2_ADAP1_ITGB8_NRCAM_CNTNAP2_AGAP3_LOXL2_MATN2_COL14A1_MTSS1_ASAP1_PTPRD_PARD3_PCDH15_HABP2_ADAM12_CDHR5_NELL1_LRRC4C_ARAP1_FAT3_CNTN5_NCAM1_MPZL2_OPCML_CLSTN3_CLEC4C_TSPAN11_PKP2_GPC6_COL4A2_NID2_NRXN3_PRTG_MEGF11_CDH3_CNTNAP4_CDH13_ITGB3_COL1A1_PTPRM_DCC_NPHS1_CLC_PSG2_PTPRT_CDH4_DSCAM_COL6A2_CELSR1 |
| GO:0048858_cell_projection_morphogenesis | 1.57E-68 | 0.0008 | EPHB2_NTNG1_LMX1A_NFASC_NRXN1_ALMS1_CTNNA2_NRP2_KLF7_CNTN4_TOP2B_EPHB1_SLIT2_UNC5C_SEMA6A_SLIT3_EGFR_SEMA3A_NRCAM_PARD3_DSCAML1_PVRL1_FEZ1_MYCBP2_NRXN3_BCL11B_IGF1R_RTN4RL1_GAS7_PRKCA_PTPRM_AFG3L2_DCC_CACNA1A_SNAP25_CDH4_APP_DSCAM |
| PANTHER_MOLECULAR_FUNCTION_Chromatin/chromatin-binding_protein | 5.44E-13 | 0.0008 | BRDT_DPF3_TOX3_L3MBTL4 |
| GO:0007420_brain_development | 1.81E-46 | 0.0008 | TP73_DAB1_LMX1A_PLXNA2_ALK_CTNNA2_CNTN4_TOP2B_SLIT2_UNC5C_LEF1_FYN_FOXP2_CNTNAP2_PRKG1_TACC2_DSCAML1_CLN5_ESR2_RORA_IGF1R_CACNA1A_CDK5RAP1_APP |
| GO:0007155_cell_adhesion | 3.21E-139 | 0.001 | COL16A1_CSF3R_PTPRF_DAB1_NEGR1_COL24A1_PTPRC_NFASC_LAMB3_NRXN1_ANTXR1_CTNNA2_PKP4_NRP2_FN1_SNED1_CNTN4_MAGI1_CNTN3_BOC_COL6A5_CLSTN2_NLGN1_PCDH7_LEF1_FAT4_CTNND2_GPR98_BTBD9_COL12A1_CD164_RADIL_ITGB8_EPDR1_EGFR_NRCAM_CNTNAP2_LOXL2_COL14A1_MTSS1_PGM5_COL27A1_LAMC3_PCDH15_CTNNA3_COL13A1_HABP2_ADAM12_CDHR5_SPON1_NELL1_FLRT1_FAT3_CNTN5_NCAM1_MPZL2_PVRL1_FEZ1_OPCML_CLSTN3_SSPN_PKP2_PLXNC1_STAB2_LMO7_ITGBL1_NID2_NRXN3_CDH3_CNTNAP4_CDH13_ITGB3_CD300A_PTPRM_MUC16_NPHS1_CD93_PTPRT_CDH4_COL20A1_APP_DSCAM_COL6A2_PARVB_CELSR1 |
| GO:0030182_neuron_differentiation | 8.54E-93 | 0.001 | TP73_EPHA2_EPHB2_OLFM3_NTNG1_LMX1A_NFASC_NRXN1_ALMS1_CTNNA2_NRP2_KLF7_CNTN4_ATP2B2_TOP2B_EPHB1_SLIT2_UNC5C_GPR98_SEMA6A_SLIT3_TULP1_CLIC5_SEMA3A_NRCAM_CNTNAP2_PARD3_PRKG1_KCNMA1_PAX6_MYO7A_DSCAML1_PVRL1_FEZ1_OPCML_PTPRR_MYCBP2_NRXN3_BCL11B_RORA_NTRK3_IGF1R_RTN4RL1_GAS7_STAT3_PRKCA_PTPRM_AFG3L2_DCC_CACNA1A_SNAP25_CDH4_APP_DSCAM |
| PANTHER_MOLECULAR_FUNCTION_Annexin | 1.37E-14 | 0.001 | PRKCE_CRTAC1_NELL1_PRKCA_FBN3 |
| GO:0022610_biological_adhesion | 3.21E-139 | 0.001 | COL16A1_CSF3R_PTPRF_DAB1_NEGR1_COL24A1_PTPRC_NFASC_LAMB3_NRXN1_ANTXR1_CTNNA2_PKP4_NRP2_FN1_SNED1_CNTN4_MAGI1_CNTN3_BOC_COL6A5_CLSTN2_NLGN1_PCDH7_LEF1_FAT4_CTNND2_GPR98_BTBD9_COL12A1_CD164_RADIL_ITGB8_EPDR1_EGFR_NRCAM_CNTNAP2_LOXL2_COL14A1_MTSS1_PGM5_COL27A1_LAMC3_PCDH15_CTNNA3_COL13A1_HABP2_ADAM12_CDHR5_SPON1_NELL1_FLRT1_FAT3_CNTN5_NCAM1_MPZL2_PVRL1_FEZ1_OPCML_CLSTN3_SSPN_PKP2_PLXNC1_STAB2_LMO7_ITGBL1_NID2_NRXN3_CDH3_CNTNAP4_CDH13_ITGB3_CD300A_PTPRM_MUC16_NPHS1_CD93_PTPRT_CDH4_COL20A1_APP_DSCAM_COL6A2_PARVB_CELSR1 |
| GO:0031175_neurite_development | 1.56E-70 | 0.001 | EPHB2_NTNG1_LMX1A_NFASC_NRXN1_CTNNA2_NRP2_KLF7_CNTN4_TOP2B_EPHB1_SLIT2_UNC5C_SEMA6A_SLIT3_TULP1_EGFR_SEMA3A_NRCAM_PARD3_PRKG1_DSCAML1_PVRL1_FEZ1_MYCBP2_NRXN3_BCL11B_RASGRF1_IGF1R_RTN4RL1_GAS7_PRKCA_PTPRM_AFG3L2_DCC_CACNA1A_SNAP25_CDH4_APP_DSCAM |
| Panther_Cadherin_signaling_pathway | 2.19E-14 | 0.001 | WNT2B_CTNNA2_LEF1_CTNNA3_WNT9B |
| GO:0005080_protein_kinase_C_binding | 1.09E-12 | 0.001 | TOP2B_NELL1_FEZ1_RBCK1 |
| GO:0008038_neuron_recognition | 7.20E-18 | 0.002 | CNTN4_SLIT2_SEMA3A_NRCAM_CNTNAP2_OPCML_APP |
| GO:0050839_cell_adhesion_molecule_binding | 1.49E-19 | 0.002 | CTNNA2_CTNNA3_PVRL1_GRIN2B_GRIN2A_CDH13_PTPRM_PTPRT |
| GO:0000902_cell_morphogenesis | 1.09E-81 | 0.002 | EPHB2_TGFBR3_NTNG1_LMX1A_NFASC_NRXN1_ANTXR1_ALMS1_CTNNA2_NRP2_KLF7_FN1_CNTN4_TOP2B_EPHB1_SLIT2_SHROOM3_UNC5C_LEF1_DAB2_SEMA6A_SLIT3_CLIC5_EGFR_SEMA3A_NRCAM_PARD3_SOX6_ZW10_DSCAML1_PVRL1_FEZ1_MYCBP2_NRXN3_BCL11B_CDC42BPB_IGF1R_RTN4RL1_GAS7_PRKCA_PTPRM_AFG3L2_DCC_CACNA1A_SNAP25_CDH4_APP_DSCAM |
| GO:0048667_cell_morphogenesis_involved_in_neuron_differentiation | 2.42E-62 | 0.002 | EPHB2_NTNG1_LMX1A_NFASC_NRXN1_CTNNA2_NRP2_KLF7_CNTN4_TOP2B_EPHB1_SLIT2_UNC5C_SEMA6A_SLIT3_SEMA3A_NRCAM_PARD3_DSCAML1_PVRL1_FEZ1_MYCBP2_NRXN3_BCL11B_IGF1R_RTN4RL1_PRKCA_PTPRM_AFG3L2_DCC_CACNA1A_SNAP25_CDH4_APP_DSCAM |
| PANTHER_BIOLOGICAL_PROCESS_Synaptic_transmission | 3.68E-34 | 0.002 | NRXN1_MOXD1_OPRM1_MAGI2_CNTNAP2_OPRK1_KCNQ3_SLC1A1_SH3GL2_DLG2_KCNMB4_KCNC2_RIMBP2_NRXN3_SH3GL3_CNTNAP4_SH3GL1 |
| GO:0008037_cell_recognition | 7.20E-18 | 0.002 | CNTN4_SLIT2_SEMA3A_NRCAM_CNTNAP2_OPCML_APP |
| GO:0048813_dendrite_morphogenesis | 1.92E-12 | 0.002 | CTNNA2_KLF7_CACNA1A_DSCAM |
| GO:0045665_negative_regulation_of_neuron_differentiation | 1.92E-08 | 0.003 | LMX1A_CNTN4 |
| GO:0030030_cell_projection_organization | 2.46E-86 | 0.003 | EPHB2_NTNG1_LMX1A_DNM3_NFASC_NRXN1_ALMS1_CTNNA2_NRP2_KLF7_SPAG16_CNTN4_FGD5_TOP2B_EPHB1_NCK1_SLIT2_UNC5C_GPR98_SEMA6A_SLIT3_TULP1_CLIC5_EGFR_SEMA3A_NRCAM_MTSS1_PARD3_PRKG1_DYNC2H1_DSCAML1_PVRL1_FEZ1_MYCBP2_NRXN3_BCL11B_RASGRF1_IGF1R_CDH13_RTN4RL1_GAS7_DNAH9_PRKCA_PTPRM_AFG3L2_DCC_CACNA1A_SNAP25_CDH4_APP_DSCAM |
| GO:0045667_regulation_of_osteoblast_differentiation | 5.85E-12 | 0.004 | TWIST2_PDLIM7_NELL1_SMAD3 |
| GO:0010565_regulation_of_ketone_metabolic_process | 6.40E-12 | 0.004 | PPARGC1A_PRKAG2_ACACB_COMT |
| GO:0007409_axonogenesis | 1.52E-58 | 0.004 | EPHB2_NTNG1_LMX1A_NFASC_NRXN1_CTNNA2_NRP2_KLF7_CNTN4_TOP2B_EPHB1_SLIT2_UNC5C_SEMA6A_SLIT3_SEMA3A_NRCAM_PARD3_DSCAML1_PVRL1_FEZ1_MYCBP2_NRXN3_BCL11B_IGF1R_RTN4RL1_PRKCA_PTPRM_AFG3L2_DCC_SNAP25_CDH4_APP |
| GO:0019900_kinase_binding | 7.99E-32 | 0.004 | PTPRC_RYR2_TOP2B_PARK2_PRKAG2_MICALCL_NELL1_FEZ1_PTPRR_SMAD3_IGF1R_STAT3_MAP2K7_RBCK1_CDK5RAP1_MAPK1 |
| GO:0015629_actin_cytoskeleton | 4.04E-49 | 0.004 | IPP_CTNNA2_MYO3B_STK17B_ABLIM2_PALLD_SORBS2_MYO10_PDLIM7_CLIC5_LANCL2_MTSS1_PGM5_DAPK1_CTNNA3_SPTBN2_MYO7A_ARHGAP32_STK38L_SPTB_MYO1E_IQGAP1_MYH11_MYO1C_GAS7_MYOM1_MYO18B |
| GO:0030424_axon | 5.26E-35 | 0.004 | NFASC_CTNNA2_NRP2_CNTN4_GRM7_SEMA6A_GRIK2_GRM1_SEMA3A_CALCR_CNTNAP2_KCNMA1_NCAM1_PVRL1_MYCBP2_DCC_APP_MAPK1 |
| GO:0022008_neurogenesis | 8.91E-108 | 0.005 | TP73_EPHA2_EPHB2_PTPRF_DAB1_OLFM3_NTNG1_LMX1A_NFASC_NRXN1_ALMS1_CTNNA2_TTL_NRP2_KLF7_CNTN4_ATP2B2_TOP2B_EPHB1_NLGN1_SLIT2_UNC5C_GPR98_SEMA6A_SLIT3_TULP1_CLIC5_FYN_EGFR_SEMA3A_NRCAM_CNTNAP2_PARD3_PRKG1_KCNMA1_TACC2_PAX6_LRRC4C_MYO7A_DSCAML1_PVRL1_FEZ1_OPCML_PTPRR_MYCBP2_ESR2_NRXN3_BCL11B_RORA_NTRK3_IGF1R_GRIN2A_RTN4RL1_GAS7_STAT3_PRKCA_PTPRM_AFG3L2_DCC_CACNA1A_SNAP25_CDK5RAP1_CDH4_APP_DSCAM_NF2 |
| GO:0032989_cellular_component_morphogenesis | 1.66E-85 | 0.005 | EPHB2_TGFBR3_NTNG1_LMX1A_NFASC_NRXN1_ANTXR1_ALMS1_CTNNA2_NRP2_KLF7_FN1_OBSL1_CNTN4_TOP2B_EPHB1_SLIT2_SHROOM3_UNC5C_LEF1_DAB2_SEMA6A_SLIT3_CLIC5_EGFR_SEMA3A_NRCAM_TXNDC8_PARD3_SOX6_ZW10_DSCAML1_PVRL1_FEZ1_MYCBP2_NRXN3_BCL11B_CDC42BPB_IGF1R_MYH11_RTN4RL1_GAS7_PRKCA_PTPRM_AFG3L2_DCC_CACNA1A_SNAP25_CDH4_APP_DSCAM |
| GO:0034703_cation_channel_complex | 2.10E-33 | 0.005 | RYR2_CACNB4_KCTD8_CACNA2D1_CNTNAP2_KCNB2_KCNQ3_PPP2R4_KCNMA1_CACNA1C_KCNMB4_KCNC2_CACNG3_KCTD1_KCNG2_CACNA1A_SNAP25 |
| GO:0005198_structural_molecule_activity | 5.65E-66 | 0.005 | COL16A1_COL24A1_LAMB3_CTNNA2_FN1_NCK1_RPL9_SORBS2_NUP155_SLIT3_COL12A1_LAMA2_LAMB1_COL14A1_PGM5_CYLC2_EPB41L4B_COL27A1_LAMC3_CTNNA3_COL13A1_NUP98_NELL1_SPTBN2_IFLTD1_BICD1_COL4A2_TUBGCP3_SPTB_MYH11_WNT9B_COL1A1_MYOM1_TUBB6_RPS28_NUP62_COL20A1_COL6A2 |
